# Synergistic Interaction of Caspofungin Combined with Posaconazole against *FKS* Wild-Type and Mutant *Candida auris* Planktonic Cells and Biofilms

**DOI:** 10.3390/antibiotics11111601

**Published:** 2022-11-11

**Authors:** Noémi Balla, Fruzsina Kovács, Bence Balázs, Andrew M. Borman, Aliz Bozó, Ágnes Jakab, Zoltán Tóth, Ola Kobaissi, László Majoros, Renátó Kovács

**Affiliations:** 1 Department of Medical Microbiology, Faculty of Medicine, University of Debrecen, 4032 Debrecen, Hungary; 2 Doctoral School of Pharmaceutical Sciences, University of Debrecen, 4032 Debrecen, Hungary; 3 UK National Mycology Reference Laboratory, Public Health England, Science Quarter, Southmead Hospital, Bristol BS10 5NB, UK; 4 Medical Research Council Centre for Medical Mycology (MRCCMM), University of Exeter, Exeter EX4 4QD, UK

**Keywords:** *Candida auris*, FIC index, Bliss independence model, combination, synergy, LIVE/DEAD, echinocandin resistance

## Abstract

*Candida auris* is a potential multidrug-resistant pathogen able to cause biofilm-associated outbreaks, where frequently indwelling devices are the source of infections. The number of effective therapies is limited; thus, new, even-combination-based strategies are needed. Therefore, the in vitro efficacy of caspofungin with posaconazole against *FKS* wild-type and mutant *Candida auris* isolates was determined. The interactions were assessed utilizing the fractional inhibitory concentration indices (FICIs), the Bliss model, and a LIVE/DEAD assay. Planktonic minimum inhibitory concentrations (pMICs) for the caspofungin–posaconazole combination showed a 4- to 256-fold and a 2- to 512-fold decrease compared to caspofungin and posaconazole alone, respectively. Sessile minimum inhibitory concentrations (sMICs) for caspofungin and posaconazole in combination showed an 8- to 128-fold and a 4- to 512-fold decrease, respectively. The combination showed synergy, especially against biofilms (FICIs were 0.033–0.375 and 0.091–0.5, and Bliss cumulative synergy volumes were 6.96 and 32.39 for echinocandin-susceptible and -resistant isolates, respectively). The caspofungin-exposed (4 mg/L) *C. auris* biofilms exhibited increased cell death in the presence of posaconazole (0.03 mg/L) compared to untreated, caspofungin-exposed and posaconazole-treated biofilms. Despite the favorable effect of caspofungin with posaconazole, in vivo studies are needed to confirm the therapeutic potential of this combination in *C. auris*-associated infections.

## 1. Introduction

*Candida auris* is an emerging pathogen, presumably related to global warming, and causes invasive infections and nosocomial outbreaks worldwide [1]. The Centers for Disease Control and Prevention (CDC) have expressed alarm that more than 90% of isolates are resistant to fluconazole, frequently accompanied by a decreased susceptibility to amphotericin B (30% resistance) and echinocandins (3 to 7% resistance) [2,3,4]. Based on isolates collected from New York and New Jersey between 2016 and 2020, echinocandin resistance increased from 0% to 4% [5]. Furthermore, a worrisome 37% increase in minimum inhibitory concentrations (MICs) to caspofungin was reported in a multicenter analysis derived from India [6]. Moreover, the majority of *C. auris* isolates are capable of biofilm development on a variety of surfaces, promoting nosocomial transmission. These sessile communities have 100–1000-fold greater MIC values than traditional antifungals; furthermore, they have higher resistance to immune response and environmental factors compared to their planktonic counterparts, which are associated with a higher ratio of clinical failures [7,8].

It is noteworthy that indwelling devices were the source of nearly 90% of *C. auris* bloodstream infections, emphasizing the clinical importance of these sessile communities [8,9]. Although echinocandins have good activity against biofilms [10], their efficacy is significantly lower against *C. auris* than against *Candida albicans* planktonic cells or biofilms [11]. Nevertheless, given the relatively low frequency of resistance to echinocandins, they are recommended as first-line agents for the treatment of invasive *C. auris* infections; however, treatment is complicated by the development of resistance in patients receiving long-term echinocandin treatment [12,13]. Echinocandin resistance is associated with mutations in the hot-spot regions of *FKS* genes, which encode the catalytic subunit of the 1,3-β-d-glucan synthase enzyme [14]. Several investigators have proposed combination-based therapeutic approaches using existing drugs to overcome the difficult-to-treat *C. auris*-related infections, including biofilm-associated cases, increasing the likelihood of therapeutic success [15,16,17,18,19]. Drug–drug combinations enhance efficacy and specificity compared to monotherapy; in addition, they can slow the evolution of resistance [19,20]. Based on previously published results, the combination of caspofungin and posaconazole has shown high efficacy against both *C. albicans* and *Candida glabrata* echinocandin-susceptible and -resistant isolates [21,22,23]. A study published by O’brien et al. (2020) examined only one posaconazole (1 mg/L) and caspofungin (4 mg/L) combination against planktonic *C. auris* cells, where synergistic interaction was observed in 13% of the tested isolates [24].

Nevertheless, whether combinations of posaconazole with echinocandins possess synergistic interactions against *C. auris*, especially against biofilms, has been poorly studied. Therefore, the major objective of our study was to evaluate the in vitro activity exerted by caspofungin and posaconazole combinations against echinocandin-susceptible (wild-type) and echinocandin-resistant (*FKS* mutant) *C. auris* planktonic cells and biofilms to provide an effective alternative therapeutic approach in biofilm-associated infections caused by this difficult-to-treat pathogen.

## 2. Results

Whole genome sequencing and *FKS1* analysis were performed for all *C. auris* isolates, and four echinocandin-sensitive isolates presented the wild-types genotype. Four isolates (Ca_1, Ca_2, Ca_3, and Ca_4) were considered to be resistant to caspofungin based on the tentative MIC breakpoint recommended by the CDC (≥2 mg/L). Two isolates (Ca_1 and Ca_2) contained the R1354H mutation in hot-spot 2 of the *FKS1* gene. Moreover, two well-described S639Y and S639P mutations were observed in the hot-spot 1 region for Ca_3 and Ca_4, respectively (Table 1).

Regarding the biofilm formation ability of the isolates, the obtained absorbance values (A_540nm_) ranged from 0.153 to 0.242 and from 0.116 to 0.170 for echinocandin susceptible and resistant isolates, respectively. The median and range of MICs for planktonic isolates and *C. auris* biofilms are presented in Table 2. Using the microdilution method, isolates were shown to exhibit pMICs for caspofungin alone from 0.5–1 mg/L and >2 mg/L for echinocandin-susceptible and echinocandin-resistant strains, respectively. In the case of posaconazole, the median pMICs ranged from 0.125 to >0.25 mg/L for both susceptible and resistant strains, respectively. The sMICs for caspofungin alone were from 32 to >32 mg/L, regardless of *FKS* phenotype. The biofilm-forming isolates exhibited sMICs for posaconazole alone from 0.25 to >2 mg/L and from 0.06 to >2 mg/L for the echinocandin-susceptible and the echinocandin-resistant strains, respectively. In the case of the echinocandin-resistant isolates, the median pMICs observed in combination showed a 4- to 256-fold reduction for caspofungin and a 2- to 256-fold reduction for posaconazole. The wild-type strains showed a reduction in MIC values for posaconazole (2- to 512-fold), while a 0 to 2-fold increase was observed in caspofungin MICs in combination with posaconazole (Table 2).

Table 3 summarizes the in vitro interactions between caspofungin and posaconazole based on the median FICIs. Antagonistic interactions were never observed (all FICIs ≤ 4). Using a two-dimensional broth microdilution checkerboard assay and FICI calculation, the nature of the caspofungin–posaconazole interaction was found to be synergistic in the case of echinocandin-resistant isolates, both for planktonic cells and biofilms, with median FICIs from 0.247 to 0.49 and from 0.091 to 0.5, respectively. In the case of the echinocandin-susceptible isolates, synergistic interactions were observed exclusively for sessile cells, with median FICIs from 0.033 to 0.375, while the nature of the interaction of their planktonic forms was indifferent, with median FICIs ranging from 1.002 to 2.001 (Table 3).

**Figure 1:**
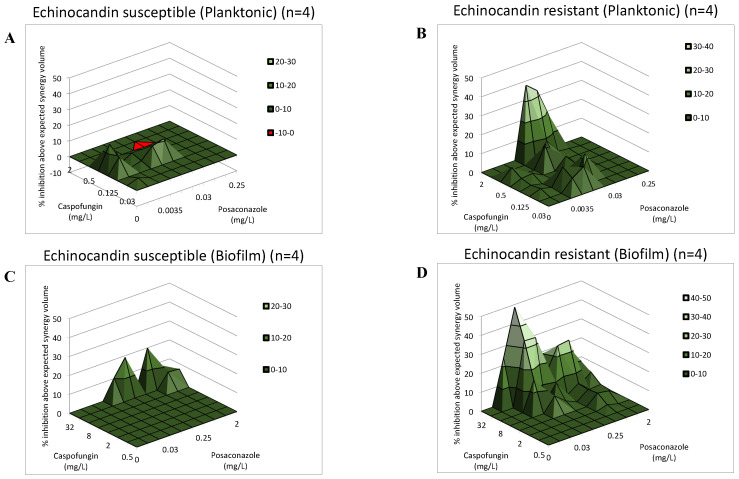
Effect of caspofungin in combination with posaconazole against *Candida auris* planktonic cells (**A**,**B**) and biofilms (**C**,**D**) using MacSynergy II analysis. Additive interactions appear as a horizontal plane at 0% inhibition. The interaction is defined as synergistic if the obtained surface is greater compared to the predicted additive surface. The volumes are calculated at the 95% confidence interval. The figures represent the cumulative synergy volume in case of four-four *FKS* wild-type (**A**,**C**) and mutant (**B**,**D**) isolates for planktonic cells and biofilms, respectively.

Figure 1 shows the dose–response surfaces for the caspofungin–posaconazole calculations with MacSynergy II. Based on the cumulative log volumes obtained, the combination of caspofungin and posaconazole produced a moderate synergy for echinocandin-susceptible strains, with 5.71 and 6.96 cumulative synergy log volumes for planktonic and sessile cells, respectively (Figure 1A,C). In the case of *FKS* mutant isolates, a strong synergy was observed, with 16.59 and 32.39 cumulative synergy log volumes for planktonic and sessile cells, respectively (Figure 1B,D).

**Figure 2:**
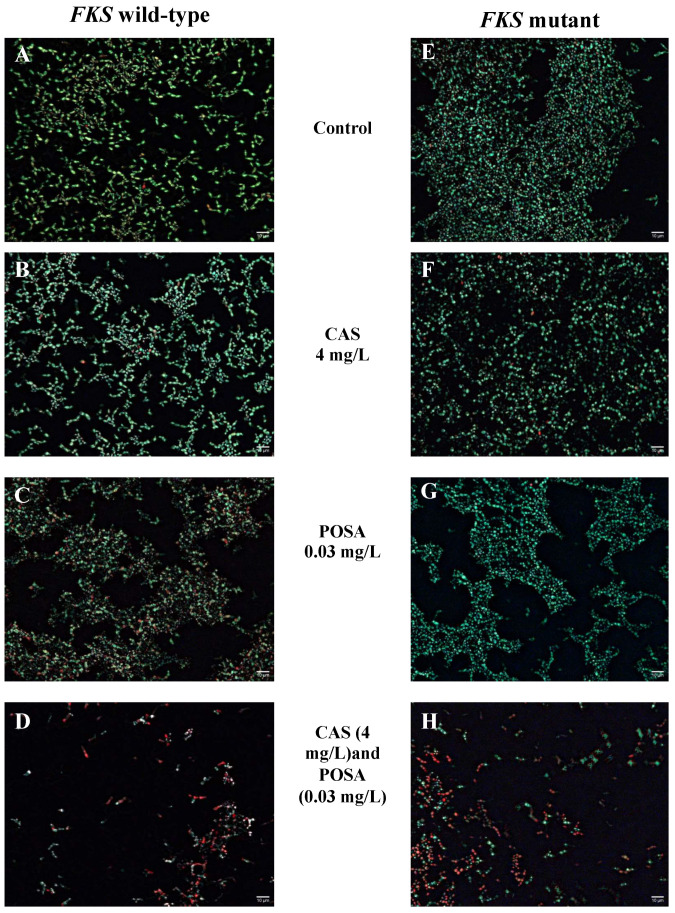
LIVE/DEAD fluorescence imaging of one *Candida auris FKS* wild-type (Ca_5) (**A**–**D**) and one *FKS* mutant (Ca_1) (**E**–**H**) representative isolates after caspofungin exposure with (**D**,**H**) and without (**B**,**F**) posaconazole. Pictures (**A**,**E**) show the untreated *C. auris* and biofilms, respectively, while picture (**C**),**G**) demonstrate the posaconazole-exposed biofilms for wild-type (**C**) and mutant (**G**), respectively. Live cells (green) and nonviable cells (red) were stained with Syto9 and propidium iodide, respectively. All images show typical fields of view. Scale bars represent 10 µm.

The strong anti-biofilm effect of the combinations was confirmed by LIVE/DEAD viability staining (Figure 2). The 4 mg/L caspofungin treatment alone did not produce remarkable cell death (Figure 2B,F). The ratio of dead cells was 0.9% and 1.4% for the echinocandin-susceptible and resistant cells, respectively (Figure 2B,F). Posaconazole exposure alone (0.03 mg/L) produce 13% and 0.2% cell death for wild-type and mutant isolates, respectively (Figure 2C,G). The combined application of caspofungin (4 mg/L) and posaconazole (0.03 mg/L) resulted in a significant total cell number reduction. The cell number was reduced by 70.7% and 80.1% for the echinocandin-susceptible and resistant cells, respectively. Moreover, the percentage of dead cells was 55.8% and 75.5% in these samples, respectively (Figure 2D,H).

## 3. Discussion

The clinical microbiology community is increasingly reporting an alarming rise in the incidence and spread of drug-resistant *C. auris*-related cases globally, which are associated with high mortality rates (30–60%) [25,26]. Several current studies have provided extensive documentation of the global antifungal resistance profiles of *C. auris* isolates to azoles and echinocandins [27,28,29]. The need for combination-based antifungal therapy against *C. auris* stems from the continuous risk of invasive infections, especially in vulnerable immunosuppressed patient populations [19,30]. Moreover, the biofilm-forming ability of this species within these susceptible patient groups further exacerbates the risk [8,9]. Despite the need for an effective and reliable approach to treatment, the clinical practice guidelines of the European Society for Clinical Microbiology and Infectious Diseases (ESCMID), the European Confederation of Medical Mycology (ECMM), and the Infection Disease Society of America (IDSA) still recommend echinocandin-based monotherapy for the majority of these infections [31,32]. The emergence of echinocandin resistance is, therefore, a relevant concern [24,33]. Based on the cutoff values suggested by the CDC, the resistance rate to echinocandins is approximately 5% [34]. Nevertheless, Kathuria et al. (2015) reported that 33 *C. auris* isolates out of 102 showed elevated MIC values (≥1 mg/L) to caspofungin in India [6]. Furthermore, none of the echinocandins had any activity in 8% of the isolates, with MICs ranging from 4 to >8 mg/L [6]. The reduced susceptibility to echinocandins is associated with mutations in the hot-spot regions of the *FKS1* or *FKS2* genes [35]. In a previous study, the poor therapeutic response was linked to the presence of the S639F *FKS1* mutation in a systemic murine model [2]. In addition, Sharma et al. (2022) observed that the *FKS1* genotype was a more accurate predictor of in vivo response than the MIC values of the isolates [14]. The *FKS* point mutations described in our study were detected previously (S639Y, S639P, and R1354H). Al-Obaid et al. (2022) reported that isolates containing the S639Y or S639P mutation in hot-spot 1 of *FKS1* exhibited reduced susceptibility to echinocandins, especially against micafungin [36]. Asadzadeh et al. (2022) also described isolates with decreased sensitivity to echinocandins carrying an R1354H mutation in hot-spot 2 of *FKS1* [37].

Several in vitro and in vivo studies on antifungal drugs have shown that combinations can broaden the coverage, increase the fungicidal effect in unresponsive cases and significantly decrease the risk of the emergence of acquired resistance [15,16,17,18]. In addition, combination-based therapeutic approaches in addition to monotherapy are also recommended in situations such as those depending on the type and site of infection and the patient’s condition [19]. Several studies have reported the negligible effect exerted by echinocandins in monotherapy against *C. auris*, both in vitro and in vivo [2,14,17,18,38]. Regarding echinocandin-based combinations, Katragkou et al. (2017) showed synergistic interactions between isavuconazole and micafungin against *C. albicans*, *C. parapsilosis*, and *C. krusei*, with the degree of synergy ranging from 1.8 to 16.7% [39]. Fakhim et al. (2017) also observed synergistic interactions between micafungin and voriconazole, with FICIs of 0.15 to 0.5 [40]. In a recent study examining 36 *C. auris* clinical isolates, synergy or partial synergy was observed in 14% and 61% of the isolates, respectively, with the combination of anidulafungin and voriconazole and in 31% and 53% of isolates, respectively, with the combination of anidulafungin and isavuconazole [41]. Caballero et al. (2021) found that isavuconazole–echinocandin combinations were more effective than monotherapy regimens [17]. These findings coincide with the results reported by Nagy et al. (2021), where caspofungin and isavuconazole showed a synergistic interaction in 61% of the tested planktonic isolates, while the ratio was 86% in the case of one-day-old biofilms [18]. Previous studies have revealed posaconazole to be the most active azole, followed by isavuconazole and itraconazole, with geometric mean MICs of 0.053 mg/L, 0.066 mg/L, and 0.157 mg/L, respectively [42]. Regarding the various azoles, Tan et al. (2021) observed the best in vitro synergy effect with minocycline against 94% of the tested strains, including *C. auris* [42]. Overall, the observed synergistic interactions may be explained by the extensive osmotic stress produced by posaconazole and caspofungin-induced impaired membrane and cell wall structure, respectively. It is noteworthy that synergy was more pronounced in the case of the *FKS* mutant isolates, especially in the case of planktonic cells. Ben-Ami et al. (2011) reported that *FKS* mutations that confer echinocandin resistance come at fitness and virulence costs, which may explain the abovementioned phenomenon [43]. The positive synergizing effect attributed to posaconazole can also be observed in more clinical cases where this drug was administered in combination-based therapies [44,45,46].

The number of in vivo experiments focusing on combination-based approaches against *C. auris* is limited. Treatment with minocycline plus posaconazole significantly increased the survival of *C. auris*-infected *Galleria mellonella*, where the survival rate was 51.7% [42]. Eldesouky et al. (2018) observed that the sulfamethoxazole–voriconazole combination administered increased the survival rate of *Caenorhabditis elegans* nematodes infected with *C. auris* by nearly 70% [47]. Nagy et al. (2021) examined the effect of caspofungin in combination with isavuconazole in vivo using an immunocompromised mouse model, where caspofungin and isavuconazole in combination was statistically superior compared with an untreated control [18].

An important strength of this study is that certain tested isolates have proven *FKS* mutations, which can be examined in terms of the planktonic and biofilm susceptibility to posaconazole and caspofungin in combination. Furthermore, whole genome sequencing was performed in the case of all isolates tested. Nevertheless, it should be highlighted that this study had a relevant limitation, namely the low number of isolates, and we could not cover all clades in terms of *FKS* mutation; therefore, we cannot conclude clade-specific consequences.

Despite these limitations, the therapeutic potential of caspofungin and posaconazole is unquestionable, having been confirmed against biofilms, especially in the case of *FKS* mutants at clinically achievable concentrations. This study suggests that the administration of caspofungin with posaconazole may help to expand potential treatment strategies. Nevertheless, combinations that proved effective in vitro needs further investigations in an immunocompromised mouse model to evaluate the pharmacokinetics/pharmacodynamics profile of these combinations and confirm their clinical applicability.

## 4. Materials and Methods

### 4.1. Isolates

Eight isolates were used, each belonging to a distinct clade of *C. auris*, referred to as South Asian, East Asian, South African, and South American clades. The characteristics of the isolates are presented in Table 1. Four out of eight isolates were *FKS* mutants, with elevated MIC values to caspofungin. The tested isolates were identified to the species level by matrix-assisted laser desorption/ionisation time-of-flight mass spectrometry (MALDI-TOF) (Microflex; Bruker Daltonics, Bremen, Germany). Clade delineation was conducted by polymerase chain reaction (PCR) amplification (GeneAmp PCR system 9700 thermocycler, Applied Biosystems, Foster City, CA, USA) and sequencing of the 28 S ribosomal DNA gene and the internal transcribed spacer region 1, as described previously [48]. Biofilm formation was assessed with the crystal violet assay, as previously described by Kovács et al. (2016) [49].

### 4.2. Whole Genome Sequencing of Isolates

Library preparation was performed using the tagmentation-based Illumina DNAFlex Library Prep kit (Illumina, San Diego, CA, USA), according to the manufacturer’s protocol. Paired-end 300 bp sequencing was executed on an Illumina MiSeq instrument. The raw sequencing reads were aligned to the *C. auris* B8441 reference genome using the Burrows–Wheeler Aligner algorithm. The genetic variants (single nucleotide polymorphisms, mutation, indel variants) were determined using the GATK algorithm. Library preparations, sequencing, and data analysis were performed at the Genomic Medicine and Bioinformatics Core Facility of the University of Debrecen, Hungary.

### 4.3. Antifungal Susceptibility Testing for Planktonic Cells

The planktonic MIC (pMIC) was determined based on the M27-A3 protocol released by the Clinical Laboratory Standards Institute (CLSI 2008) [50]. Susceptibility to caspofungin pure powder (Merck, Budapest, Hungary) and posaconazole pure powder (Merck, Budapest, Hungary) was determined in RPMI-1640 (with l-glutamine and without bicarbonate, pH 7.0, and with MOPS; Merck, Budapest, Hungary). The drug concentrations ranged from 0.0009 to 0.25 mg/L for posaconazole and from 0.03 to 2 mg/L for caspofungin. The pMICs were determined as the lowest antifungal concentration that exerts at least 50% growth inhibition compared with the untreated growth control and are presented as the median value of three independent experiments per isolate. *Candida parapsilosis* ATCC 22019 and *Candida krusei* ATCC 6258 were used as the quality-control strains.

### 4.4. Biofilm Development

One-day-old biofilms were prepared as described previously [18,49]. Briefly, following 48 h culturing on Sabouraud dextrose agar (Lab M Ltd., Bury, UK), *C. auris* cells were harvested by centrifugation (3000× *g* for 5 min), washed three times in sterile physiological saline, and the final density of the inoculums was adjusted in RPMI-1640 broth to 1 × 10^6^ cells/mL using a Burker’s chamber (Hirschmann Laborgeräte GmbH & Co. KG, Eberstadt, Germany). Afterward, 100 µL aliquots were inoculated onto flat-bottom 96-well sterile microtitre plates (TPP, Trasadingen, Switzerland) and incubated statically in darkness at 37 °C for 24 h.

### 4.5. Assessment of Antifungal Susceptibility for Biofilms

The caspofungin concentrations for the biofilm MIC (sMIC) determination ranged from 0.5 to 32 mg/L, while the examined posaconazole concentrations ranged from 0.007 to 2 mg/L. The biofilms were washed three times with sterile physiological saline. After incubation at 37 °C for 24 h, the biofilms were washed with sterile physiological saline, and an XTT-assay was performed, as described previously [18,49,51]. The change (%) in metabolic activity was calculated based on absorbance (*A*_492nm_) by using a Multiscan Sky Microplate Spectrophotometer (Thermo Fisher Scientific, Waltham, MA, USA), as:

100% × (*A*_well_ − *A*_background_)/(*A*_drug-free well_ − *A*_background_)

The *A*_background_ corresponds to 100 µL drug-free and biofilm-free XTT-solution. The sMICs were defined as the lowest drug concentration resulting in at least a 50% metabolic activity decrease compared with the untreated control cells [18,49,51] and are presented as the median value of three independent experiments per isolate.

### 4.6. Assessment of Synergy between Caspofungin and Posaconazole

The drug–drug interactions between caspofungin and posaconazole were assessed by the two-dimensional checkerboard broth microdilution assay, as previously described [18,49,51,52,53]. Planktonic and sessile cells were prepared with 2 × 10^4^ cells/mL and 1 × 10^6^ cells/mL, respectively, containing different concentrations of each drug combination. The concentrations tested corresponded to the values described in the susceptibility experiments. Afterward, the plates were incubated for 24 h at 37 °C. The data obtained from the checkerboard tests were evaluated by the fractional inhibitory concentration index (FICI), which was expressed as:

ΣFIC = FIC_A_ + FIC_B_ = [(MIC_A_^comb^/MIC_A_^alone^)] + [(MIC_B_^comb^/MIC_B_^alone^)]

where MIC_A_^alone^ and MIC_B_^alone^ are the MICs of drugs A and B when used alone, and MIC_A_^comb^ and MIC_B_^comb^ are the MICs of drugs A and B in combination at isoeffective combinations, respectively [18,49,51,52,53]. The FICIs were determined as the lowest ΣFIC. MICs of the drugs alone and those of all isoeffective combinations were determined as the lowest concentration resulting in at least a 50% reduction in turbidity and metabolic activity compared with the untreated control cells for planktonic and sessile populations, respectively. FICIs were determined in three independent experiments and are expressed as the median value. A synergistic interaction was defined as FICI ≤ 0.5, while 0.5 < FICI ≤ 4 was considered to be an indifferent interaction, and FICI > 4 was considered to be an antagonistic interaction [18,49,51,52,53].

To further evaluate the nature of the caspofungin and posaconazole interactions, MacSynergy II analysis was applied, which employs the Bliss independence algorithm in a Microsoft Excel–based interface to assess the nature of interactions [18,51,54,55,56]. This algorithm calculates the difference (ΔE) in the predicted percentage of growth (E_ind_) and the experimentally observed percentage of growth (E_exp_) to define the interaction of the drugs used in combination. The MacSynergy II model expresses interaction volumes and determines positive volumes as synergistic and negative volumes as antagonistic [18,51,54,55,56]. The E values of all combinations are presented on the *z*-axis in the three-dimensional plot. Synergy or antagonism is significant if the interaction log volumes are >2 or <2, respectively, while log volume values between >2 and 5 correspond to minor synergy, between >5 and 9 shows moderate synergy, >9 shows strong synergy, and the negative values correspond to minor, moderate and strong antagonistic interaction, respectively [18,50,53,54,55]. The synergy volumes were calculated at the 95% confidence level.

### 4.7. Biofilm Viability Assay

The effect of the combinations on biofilm viability was examined using the LIVE/DEAD^®^ BacLight™ assay against all of the isolates tested, and pictures from the one-one representative echinocandin-susceptible (strain Ca_5) and resistant (Ca_1) isolates were presented. One-day-old biofilms were grown on the surface of a 4-well Permanex slide (Lab-Tek^®^ Chamber Slide™ System, VWR, Debrecen, Hungary). The preformed biofilms were washed three times with sterile physiological saline, and various drug concentrations, chosen based on the checkerboard results, were added to the samples as follows: 4 mg/L caspofungin, 0.03 mg/L posaconazole, and 4 mg/L caspofungin combined with 0.03 mg/L posaconazole. Following 24 h of antifungal treatment, the sessile cells were washed with sterile physiological saline, and the ratio of viable and dead cells was evaluated using the fluorescent LIVE/DEAD^®^ BacLight™ viability kit (ThermoFisher scientific, USA), as described in our previous works [51,56]. The biofilms were exposed to Syto 9 (3.34 mM solution in DMSO) and propidium iodide (20 mM solution in DMSO) for 15 min in darkness at 37 °C to examine viable and dead *Candida* cells, respectively. The fluorescent cells were studied with a Zeiss AxioSkop 2 mot microscope (Jena, Germany) coupled with a Zeiss AxioCam HRc camera (Jena, Germany). The analysis of the images was performed using Axiovision 4.8.2 (Jena, Germany). The digital images were obtained using 40×/0.75 Zeiss Plan-Neofluar objective coupled with 1× C-mount. Further picture analysis and the calculation of the percentage of dead cells was performed using ImageJ software (version: 2.9.0/1.53t) (Fiji, ImageJ, Wayne Rasband National Institutes of Health). All pictures were changed to 8-bit grayscale with background noise subtracted, after which the threshold was defined [56].

## 5. Conclusions

In conclusion, our results clearly demonstrate the synergistic interactions of caspofungin in combination with posaconazole against *C. auris*, especially against biofilms. This study has the potential to be a starting point for further studies exploring the in vivo and clinical impact of these combinations against *C. auris*.

## Figures and Tables

**Table 1:** Characteristics of *Candida auris* isolates used in this study.

Isolates	Clade	Isolation Source	*FKS* Mutations
Ca_1	South Asian	wound swab	HS1 WT HS2 R1354H
Ca_2	South Asian	perianal swab	HS1 WT HS2 R1354H
Ca_3	South Asian	Central line	HS1 S639Y HS2 WT
Ca_4	South Asian	wound swab	HS1 S639P HS2 WT
Ca_5	South Asian	Unknown	HS1 WT HS2 WT
Ca_6	East Asian	Unknown	HS1 WT HS2 WT
Ca_7	South African	Tracheostomy	HS1 WT HS2 WT
Ca_8	South American	Blood	HS1 WT HS2 WT

HS1 corresponds to hot-spot 1, HS2 corresponds to hot-spot 2, WT corresponds to wild-type.

**Table 2:** Minimum inhibitory concentrations (MICs) of caspofungin alone and in combination with posaconazole against *Candida auris* planktonic cells and biofilms.

Isolates	Planktonic Cells Median MIC (Range) of Drug Used (50% OD_492nm_ Reduction in Turbidity)				Biofilms Median MIC (Range) of Drug Used (50% OD_492nm_ Reduction in Metabolic Activity)			
	Alone		In Combination		Alone		In Combination	
	Caspofungin (mg/L)	Posaconazole (mg/L)	Caspofungin (mg/L)	Posaconazole (mg/L)	Caspofungin (mg/L)	Posaconazole (mg/L)	Caspofungin (mg/L)	Posaconazole (mg/L)
Ca_1	>2 ^a^	>0.25 ^b^	1 (0.03–1)	0.002	>32 ^c^	>2 ^a^ (1–>2)	0.5	0.015
Ca_2	>2 ^a^	0.25	1	0.002	>32 ^c^	0.06	2	0.015
Ca_3	>2 ^a^	0.125 (0.06–0.125)	1 (1–2)	0.008	>32 ^c^	0.06	8 (8–16)	0.008 (0.015–0.008)
Ca_4	>2 ^a^	0.125	0.03	0.015 (0.008–0.03)	32 (32–>32 ^c^)	1 (0.125–1)	0.5 (0.5–1)	0.008
Ca_5	0.5	0.125	0.5 (0.25–0.5)	0.06	>32 ^c^	>2 ^a^	8	0.5
Ca_6	0.5	>0.25 ^b^	1	0.0009	32 (32–>32 ^c^)	>2 ^a^	0.5	0.008
Ca_7	0.5	>0.25 ^b^	0.5	0.0009	>32 ^c^	0.25 (0.25–0.5)	0.5	0.008
Ca_8	1	>0.25 ^b^	1	0.0009	>32 ^c^	1	2 (1–2)	0.06

^a^ MIC is off-scale at >2 mg/L, 4 mg/L (one dilution higher than the highest tested concentration) was used for FICI analysis. ^b^ MIC is off-scale at >0.25 mg/L, 0.5 mg/L (one dilution higher than the highest tested concentration) was used for FICI analysis. ^c^ MIC is off-scale at >32 mg/L, 64 mg/L (one dilution higher than the highest tested concentration) was used for FICI analysis.

**Table 3:** In vitro interactions by FIC indices (FICI) of caspofungin in combination with posaconazole against *Candida auris* planktonic cells and biofilms.

Isolate	Planktonic Cells		Biofilms	
	FICI		FICI	
	Median (Range) of FICI	Interaction	Median (Range) of FICI	Interaction
Ca_1	0.31 (0.31–0.37)	Synergy	0.155 (0.061–0.1876)	Synergy
Ca_2	0.37 (0.37–0.49)	Synergy	0.5 (0.375–0.75)	Synergy
Ca_3	0.49 (0.5–0.56)	Synergy	0.5 (0.5–0.75)	Synergy
Ca_4	0.247	Synergy	0.091 (0.038–0.315)	Synergy
Ca_5	1.24 (1–1.24)	Indifferent	0.375 (0.25–0.5)	Synergy
Ca_6	2.001	Indifferent	0.033	Synergy
Ca_7	2.001	Indifferent	0.0378 (0.031–0.038)	Synergy
Ca_8	1.002	Indifferent	0.25 (0.185–0.281)	Synergy

## Data Availability

The data shown and discussed in this paper have been deposited in the NCBI GenBank with the following BioProject no.: PRJNA865124.
